# Perioperative Sensor and Algorithm Programming in Patients with Implanted ICDs and Pacemakers for Cardiac Resynchronization Therapy

**DOI:** 10.3390/s21248346

**Published:** 2021-12-14

**Authors:** Alexander Niedermeier, Laura Vitali-Serdoz, Theodor Fischlein, Wolfgang Kirste, Veronica Buia, Janusch Walaschek, Harald Rittger, Dirk Bastian

**Affiliations:** 1 Faculty of Medicine, Friedrich-Alexander-University Erlangen-Nuernberg (FAU), 91054 Erlangen, Germany; alex.niedermeier@web.de; 2 Department of Cardiology, Klinikum Fuerth, Teaching Hospital of Erlangen-Nuernberg University, 90766 Fuerth, Germany; veronica.buia@klinikum-fuerth.de (V.B.); janusch.walaschek@klinikum-fuerth.de (J.W.); Harald.rittger@klinikum-fuerth.de (H.R.); dirk.bastian@klinikum-fuerth.de (D.B.); 3 Department of Cardiac Surgery, Cardiovascular Center, Klinikum Nuernberg—Paracelsus Medical University, Breslauer Str. 201, 90419 Nuremberg, Germany; Theodor.fischlein@klinikum-nuernberg.de; 4 Outpatient Clinic for Cardiology and Diabetes, 91126 Schwabach, Germany; kirstewolfgang@yahoo.de

**Keywords:** implantable cardioverter defibrillator, ICD, sensors, sudden cardiac death, heart failure, defibrillator shock, cardiac resynchronization therapy, electromagnetic interference

## Abstract

Background: ICDs and pacemakers for cardiac resynchronization therapy (CRT) are complex devices with different sensors and automatic algorithms implanted in patients with advanced cardiac diseases. Data on the perioperative management and outcome of CRT carriers undergoing surgery unrelated to the device are scarce. Methods: Data from 198 CRT device carriers (100 with active rate responsive sensor) were evaluated regarding perioperative adverse (device-related) events (A(D)E) and lead parameter changes. Results: Thirty-nine adverse observations were documented in 180 patients during preoperative interrogation, which were most often related to the left-ventricular lead and requiring intervention/reprogramming in 22 cases (12%). Anesthesia-related events occurred in 69 patients. There was no ADE for non-cardiac surgery and in pacemaker-dependent patients not programmed to an asynchronous pacing mode. Post-operative device interrogation showed significant lead parameter changes in 64/179 patients (36%) requiring reprogramming in 29 cases (16%). Conclusion: The left-ventricular pacing lead represents the most vulnerable system component. Comprehensive pre and post-interventional device interrogation is mandatory to ensure proper system function. The type of ICD function suspension has no impact on each patient’s outcome. Precautionary activity sensor deactivation is not required for non-cardiac interventions. Routine prophylactic device reprogramming to asynchronous pacing appears inessential. Most of the CRT pacemakers do not require surgery-related reprogramming.

## 1. Introduction

**Figure 1:**
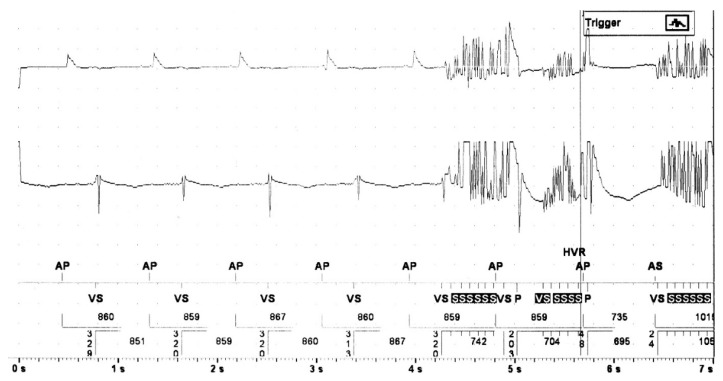
Example of electromagnetic interference due to monopolar electrocautery. Both the atrial (fist line) and ventricular EGM (middle line) show pulsed artefacts in a patient with implanted conventional dual chamber pacemaker undergoing repair of the ascending aorta.

Patients with cardiac implantable electronic devices (CIED) undergoing non-device associated surgery require an individual peri-interventional management, in particular with regard to possible side effects of electromagnetic interferences (EMI, Figure 1) [1].

Among all CIED carriers, patients with implanted systems for cardiac resynchronization therapy (CRT) form a specific subpopulation requiring particular perioperative attention and care for several reasons. Compared with conventional implanted cardioverter defibrillators (ICDs) or pacemakers, the major indication for CRT is symptomatic drug refractory heart failure [2] that is per se a known independent risk factor for perioperative complications [3].Furthermore, CRT carriers are often older with frequent comorbidities such as atrial fibrillation (AF), diabetes, and chronic kidney disease (CKD) [4], which contribute to a worse outcome [5,6,7]. In addition to the underling cardiac disease, the perioperative risk may be affected significantly by the device itself, because ICDs and pacemakers implanted for CRT represent the most complex type of CIEDs. The software encloses a large variety of different sensors, automatic algorithms, and programming features, and it may differ depending on the manufacturer [8,9,10]. The biventricular hardware configuration with a third lead wedged in an epicardial coronary sinus side branch is associated with higher complication rates in the long term compared with conventional devices [11,12,13,14]. Although current practice advisories and recommendations for the management of CIED carriers refer also to biventricular devices, clinical data on the perioperative management and outcome of CRT patients are limited. The EVINCE-CRT study (Perioperative Management Evaluation in Patients With Implanted Cardiac Electronic Devices-CRT) aims to evaluate the perioperative management and outcome of patients with implanted CRT devices undergoing non-CIED-related surgery or catheter interventional procedures with special focus on the programming of sensors and automatic algorithms.

## 2. Patients and Methods

The study analyzes comprehensive data from 198 non-CIED-associated surgical or catheter-interventional procedures performed in patients implanted with CRT-ICDs (CRT-D) or a CRT-pacemaker (CRT-P) between 2008 and 2021.

Patients included in the study form a predefined subpopulation of a large observational registry that prospectively enrolls adult patients (>18 years) with CIEDs evaluating the perioperative management at two German centers (Klinikum Fuerth and Klinikum Nuernberg) since 2008 (“EVINCE-CRT”). At present, the EVINCE study contains data on 1085 surgical and catheter-interventional procedures. The study was performed according to the requirements for obtaining the medical degree (Dr. med), applies to the ethical standards contained in the Declaration of Helsinki, and consent to the procedure was obtained from each patient. The trial protocol was approved by the responsible ethics committee (Friedrich Alexander University Erlangen-Nuremberg, 385_18 Bc) and is registered at ClinicalTrials.gov PRS (NCT04331249).(accessed on 13 December 2021).

Except for cases of emergency intervention, the CIED was interrogated pre- and post-operatively. The type of pre-interventional device programming was not predefined and left to the decision of the cardiologist performing the interrogation in consultation with the operator and/or anesthesiologist aiming to limit reprogramming to the minimum. The sensor for rate-adaptive pacing was deactivated for cardiac interventions and at the operator’s or anesthesiologist’s request. For ICDs without suspension of the antitachyarrhythmia function by programming, the type of magnet reaction was verified prior to surgery to ensure appropriate behavior. All other automatic algorithms were left active if applicable (e.g., automatic sensing, automatic capture control (ACC), auto-PVARP, noise detection, lead integrity detection, etc.). In pacemaker-dependent patients, the noise interference mode had to be programmed to “asynchronous” pacing. In every case, the operator and anesthesiologist were informed regarding the device function and parameters (e.g., pacemaker dependency, mode programmed, lowest pacing rate, sensor and ICD function programming). To reduce EMI, the recommendation was to prefer bipolar electrocautery and to take care on the correct positioning of the neutral electrode to keep the electrocautery device away from the pacemaker and to use only brief bursts with the lowest amplitude possible [15]. Peri-interventional electrocardiographic and hemodynamic monitoring was obligatory as well as the immediate availability of an external pacer/defibrillator.

All adverse events (AE), adverse device effects (ADE), and anesthesia-related observations and incidents/events (ARE) [16] were documented and classified according to the ISO/DIS 14,155 standard with respect to their relationship to the surgical or catheter interventions [17,18]. The ASA physical status (PS) was classified according to [19].

Primary safety outcome was the number and type of perioperative ADE.

Further, data from pre-interventional CIED interrogation including programming of sensors, automatic algorithms and antitachycardia function, peri-interventional data (e.g., type of intervention, surgery above or below the umbilicus, electrocautery, anesthesiology techniques, any ARE) and data from post-interventional CIED interrogation were evaluated in relation to the patient’s outcome.

Predefined outcome measures were a post-procedural pacing threshold increase >50% compared with the pre-procedural threshold (given a safety margin of 100%), a post-procedural sensing decrease by >50% compared with the pre-procedural sensing, and a post-procedural lead impedance different by >25% compared with the pre-procedural impedance.

Pacemaker dependency was defined as a history of syncope, atrioventricular nodal ablation, and/or an intrinsic rhythm < 40 beats/min [20] during device interrogation with a subgroup of patients without escape rhythm > 30 beats/min.

The statistic has a descriptive character. Clinical and device-technical parameters are presented as means ± standard deviation. Continuous variables were compared by Student’s T-test or the Mann–Whitney U test for normally and non-normally distributed data, respectively. The χ2 test or Fisher exact test was used for nominal variables. Correlation of continuous variables was assessed by Pearson correlation coefficient. A *p* value < 0.05 was considered statistically significant. The analysis was performed with SPSS, version 20 (SPSS Inc., Chicago, IL, USA).

## 3. Results

### 3.1. Baseline Characteristics and CRT Devices

One hundred ninety-eight invasive procedures were performed in 134 patients implanted with a CRT-D (68%) and 64 patients with a CRT-P at a mean age of 72.2 ± 9.6 years (range 44–93 years). Nearly every fourth subject (24%) was ≥80 years.

Baseline characteristics are reported in Table 1.

Information on CRT indication was available in 152 (77%) patients. Main indications were chronic heart failure (107, 70.4%), left bundle branch block (LBBB, 80, 52.6%), high-degree atrioventricular (AV) block (AVB) with indication for ventricular pacing (63, 40.9%), and atrioventricular junction (AVJ) ablation (13, 6.6%). The ICD was implanted in 97 cases (80.8%) for primary prevention of sudden cardiac death. Underling cardiomyopathy could be evaluated in 130 ICD recipients and was ischemic or non-ischemic in 61 (47%) and 69 (53%) cases, respectively.

Information on the left ventricular (LV) ejection fraction (LV-EF) was available in 97 cases with a mean of 30.7 ± 9.5%. Seventy-seven interventions (79.4%) were performed in patients with an LV-EF ≤ 35%. The PS was classified as ASA II in 29 (14.7%), ASA III in 141 (71.6%), and ASA IV in 27 (13.7%) patients, respectively.

Fifty-one different types of CRT devices manufactured by Biotronik (Berlin, Germany), Guidant/Boston Scientific (Marlborough, MA, USA), Medtronic (Minneapolis, MN, USA), and St. Jude Medical/Abbott (Lake Forest, IL, USA) were investigated (Table 2) with 114 systems (58%) implanted outside the study centers. In 51 patients, the primary device had already been replaced at least once. In 16 cases (8.4%), the current CRT system was implanted as an upgrade of a former single- or dual-chamber device.

### 3.2. Pre-Interventional CRT Interrogation, Sensor, and Algorithm Programming

**Figure 2:**
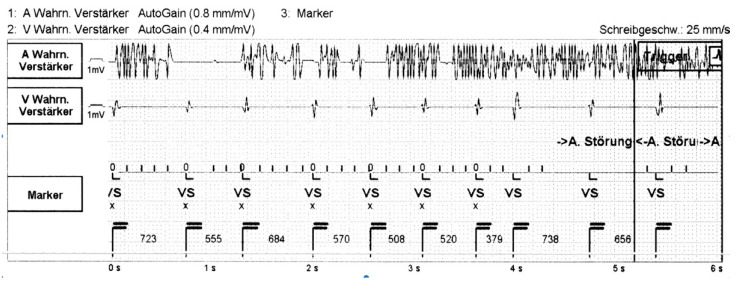
Right atrial lead defect. Artifacts in the atrial EGM (A, first line) documented during preoperative interrogation of a CRT device with an already known isolation defect of the atrial lead (inactivated, impedance < 100 Ω). There is no noise on the ventricular (V) lead channel (middle line). Paper speed 25 mm/s. VS. = ventricular sensed event. “A. Störung” = atrial noise detection.

One hundred and eighty patients (91%) underwent pre-interventional device interrogation. A device ID card was presented by 145 patients (81%) with large variations regarding the content herein. In 21 out of 163 patients (10.6%) with available information, the time interval to the last preceding device interrogation was longer than six months. There were known device-associated adverse findings in 39/145 (27%) patients (Figure 2), most often an increased LV-pacing threshold > 2 Volt (14/145 patients, 9.6%) and low phrenic nerve capture threshold in three cases. In five subjects, the LV lead was inactive (Table 3).

Eighty patients (44%) were in AF or atrial flutter. Atrial mode switch and episodes of any type of ventricular tachyarrhythmia were documented in 42 and 33 patients, respectively. Information on biventricular pacing delivery was available in 162 patients with a mean of 90.4 ± 19.5%. Thirty-eight subjects (23.5%) had <90% biventricular pacing, among them 12 (31%) patients in sinus rhythm (SR). In four cases, the remaining battery longevity was ≤6 months.

Thirty-nine new device-related observations were reported during the pre-interventional interrogation, 28 of them requiring a reprogramming or intervention in 22 patients (12%) to ensure proper device function but in no case for vital indication (Table 4). In 13 cases, a technical finding was related to the LV lead. Of note, in three patients, the programmed LV output was less than the measured pacing threshold. The LV lead had to be inactivated in two cases. In two patients with scheduled ventricular tachycardia (VT) catheter ablation, the clinical VT was induced during the LV pacing threshold test.

On request of the operator and/or anesthesiologist, the antitachycardia ICD function was suspended in 60 out of 134 CRT-D carriers. Pacing mode was changed to DOO or VOO on request in only two cases for renal and rectal surgery. The pacemaker function was suspended (OVO/ODO or “off”) in five cases for catheter ablation procedures. An atrial tracking mode was switched to a non-tracking mode due to AF undersensing in one case, and in one patient with VVI pacemaker syndrome in SR but with no atrial lead, the functionality was changed to back-up pacing only.

In nine out of 109 patients with initially active accelerometer sensor, the rate response was deactivated for cardiac intervention. Proper function provided, all automatic functions for sensing and pacing were left active irrespective of pacemaker dependency.

In total, the device was reprogrammed in 85 patients (42.9%).

### 3.3. Surgery and Peri-Interventional Findings

Detailed information on the type of intervention and anesthesia were available in 197 cases (99.5%). There were 150 open surgical procedures, 29 endoscopic and 15 catheter-based interventions performed in 67 cases (34%) above and in 130 patients below the level of the umbilicus (Table 5 and Table 6).

Any ARE was observed in 69 patients with hypotension in most cases (Table 7). Patients with ARE were older (74.9 ± 9.3 vs. 70.7 ± 9.6 years, *p* = 0.004) and had lower LV-EF (27.5 ± 6.7% vs. 32.3 ± 10.4%, *p* = 0.022).

There was no impact of the site of surgery (above or below the umbilicus) on the number or type of ARE (Table 8).

Compared with surgery below the umbilicus, supraumbilical interventions had a significant correlation with peri-interventional changes in the impedance values of the RA (*p* < 0.000) and LV lead (*p* = 0.001) in the general population but no impact on other measurements or need for post-procedural programming.

Six events were considered potential ADE according to the anesthesiologic report. In two cases, device-associated tachycardia was suspected; however, post-interventional CRT-P interrogation revealed paroxysmal atrial tachyarrhythmia as the underlying mechanism. Another observation was bradycardia with a heart rate, which corresponded to the programmed lower rate limit (50 beats/min). Two further suspected ADEs resulted in fact from proper CRT-D behavior. In the first case, the antitachycardia function was not suspended, no magnet was used for arteriovenous shunt surgery, and the ICD delivered an appropriate shock to terminate intraoperative VT. The second case was likewise appropriate shock delivery for VT immediate after ICD reprogramming following peritoneal shunt surgery.

Nine patients (age 68 ± 15 years, LV-EF 23 ± 9%, five CRT-D) underwent elective heart surgery including one case with transfemoral TAVI. Six patients were pacemaker dependent. The ASA PS was 3 and 4 in six and three patients, respectively. The rate response was inactive in all patients. Among these patients, one ADE was confirmed and caused by diaphragmatic stimulation during cardioplegia occurring in a pacemaker-dependent CRT-D carrier undergoing coronary bypass surgery. Post-operative device interrogation showed a loss of capture for the atrial and LV lead, resulting in clinical pacemaker syndrome and requiring system revision. A further true ADE was not reported, but in the CRT-D device memory of a patient undergoing mitral valve reconstruction and magnet application, there were two appropriate shock deliveries for (induced) ventricular fibrillation documented.

Thirty-five patients (27 with CRT-D) underwent surgery for neoplastic disease without a significant increase in ARE compared to the general population (*p* = 0.165).

There was no intraoperative death.

### 3.4. ICD Antitachycardia Function Suspension

The antitachycardia function was suspended in 60 CRT-D by programming and in 59 patients using a magnet. There was no significant correlation between these two approaches regarding number of ARE (*p* = 0.138) or any change of electrode parameters (*p* = 0.158). There was no inappropriate ICD therapy during magnet application for non-cardiac surgery. Fifteen patients underwent surgery without ICD inactivation. Compared with inactivated devices, there was no difference regarding ARE (*p* = 0.116) or lead parameter changes (*p* = 0.172).

### 3.5. Sensor Programming

One hundred patients (47 CRT-P, 53 CRT-D) underwent surgery with active rate responsive accelerometer sensor. In the subgroup with an inactive sensor, there were more patients with implanted ICD (17 CRT-P, 81 CRT-D, *p* = 0.000), in SR (*p* = 0.000) who were not pacemaker-dependent (*p* = 0.001), but both populations were not different regarding LV-EF (*p* = 0.249), biventricular pacing (*p* = 0.064), and ASA PS (*p* = 0.418). There was no difference regarding the number and type of perioperative ARE. In particular, there were no confirmed inappropriate sensor-mediated tachycardias during or after non-cardiac interventions.

### 3.6. Automated Capture Control and Pacemaker Dependency

Eighty-one patients (41%) met the formal criteria for pacemaker dependency with 62 patients showing no escape rhythm during device interrogation.

Atrial, RV, or LV ACC was active in 34, 61, and 24 patients, respectively. At least one automatic algorithm for ventricular ACC was active in 75 patients (37.9%), among them 26/81 (32.1%) pacemaker-dependent patients.

There was no correlation between any active ventricular ACC, any peri-interventional change of lead parameters (*p* = 0.082), or any ARE (*p* = 0.641) in the general population and among pacemaker-dependent patients (*p* = 0.388 for any change of lead parameters, *p* = 0.188 for any ARE).

### 3.7. Oversensing and Potential EMI

Artifacts related to myopotential oversensing were detected in a patient without in-house pre-interventional device interrogation with a magnet inactivated CRT-D, undergoing uneventful implantation of an endovascular popliteal stent-prothesis without cauterization. During the post-operative follow-up, the ventricular sensitivity was reprogrammed, and the LV lead had to be inactivated due to a low phrenic nerve capture threshold.

There was only one CRT-D patient with documented non-physiologic short VV intervals potentially caused by EMI detected during neck surgery for local abscess using monopolar electrocautery. In this case, antitachycardia therapy had been deactivated before surgery, and no ADE occurred.

The type of electro-cauterization used was categorized into two groups: monopolar alone or combined use with bipolar together and bipolar cauterization only. There was a significant association between a decrease in atrial lead impedance and the use of monopolar cautery (*p* = 0.02). No significant impact on any other lead parameter change was found.

### 3.8. Post-Interventional Findings and Outcome

Postoperative device interrogation showed significant changes in sensing, pacing threshold, or impedance in 64/179 patients (35.8%) requiring appropriate programming in 29 cases (16%, Table 9 and Table 10). In two patients, the LV lead had to be inactivated for persistent high pacing threshold. One further patient had to undergo system revision due to loss of left ventricular capture following cardiac surgery (re-mitral valve replacement). In one patient, the pacing mode was changed to AAIR because of the increased atrial and right ventricular pacing capture threshold after radiofrequency energy catheter ablation for VT.

In summary, there were four confirmed ADEs, which are all associated with cardiac interventions.

Comparing the postoperative lead specific sensing, pacing threshold, or impedance with the preoperative values, we documented a significant decrease in the sensing and impedance of all the leads (Table 10).

Four patients died after surgery. In all cases, mortality was related to the severe underlying disease. There was no association with any device malfunction (Table 11).

### 3.9. Longer-Term Follow-Up

Data on longer-term follow-up were available in 52 patients (26%) with device interrogation after 14.9 ± 22.28 months following surgery. One CRT-D was explanted due to infection and later replaced by a subcutaneous ICD. Increased RV pacing threshold was documented in one case. There was no additional surgery-associated adverse observation.

## 4. Discussion

In addition to the type of surgery, the peri-interventional risk of patients with CIEDs depends predominantly on the underling disease but also on the implanted device [1,21]. Adverse outcomes may be clinical (e.g., hypotension, brady- or tachyarrhythmia, death) or CIED associated such as hardware damage, changed pacing behavior (e.g., sensor-induced tachycardia), or shock delivery caused either by EMI or mechanically [17].

Since the first reports on perioperative ADEs and EMI, there has been a tremendous evolution in both fields, the treatment of the underlining diseases, and the device technology. The development of modern drugs as well as CIED-based therapy such as CRT, cardiac contractility modulation, or conductive system pacing has led to a significant reduction of morbidity and mortality of heart failure [22,23,24,25]. In current CIEDs, modern programming strategies, filters, shielding and noise detection algorithms provide improved protection against EMI and inappropriate therapies [20,26,27]. However, on the other hand, modern device technology has become much more dependable and complex by the introduction of a wide range of programmable and/or automatic sensors and features [28].

Based on often small observational studies and historic case reports, current practice advisories on the management of ICD and pacemaker carriers refer also to biventricular devices [17]. However, patients with implanted biventricular CIEDs for CRT are largely underrepresented in previous trials, and recommendations on the need for preoperative device interrogation and reprogramming are often equivocal. Compared with “conventional” pacemaker carriers, indication for CRT implantation in advanced, and drug-refractory chronic cardiac disease [1,2] is per se an independent risk factor for perioperative complications [3]. In addition, CIEDs for CRT represent the currently most complex devices regarding both hardware and software with a reported complication rate of up to 12.4% at 6 months follow-up [29]. It has been stated that “The complexity of cardiac generators limits generalizations…” [30] (p. 261) and therefore, observations made from patients with conventional single- or dual-chamber devices may not necessarily be transferrable one-to-one to CRT carriers.

To add clinical evidence, the EVINCE-CRT registry evaluated comprehensive data from 198 patients with implanted CRT devices undergoing non-device-related surgery and catheter interventions with the following main findings:

(1) There was no device-associated peri-interventional mortality.
(2) In CRT carriers, the rate of pre- and post-interventional reprogramming required to ensure proper device function is high. The CRT-specific left-ventricular pacing lead represents the most vulnerable system component being responsible for the majority of perioperative interventions.
(3) For non-cardiac surgery, there was no perioperative device-associated adverse event.
(4) Cardiac interventions may be associated with an increased risk for lead damage in complex devices, but this finding requires further evaluation.
(5) Neither programming of sensors and automatic algorithms nor the type of antitachycardia function suspension in CRT-ICDs had an impact on perioperative adverse events, lead parameters, and outcome.
(6) The majority of pacemakers for CRT did not require any programming.
(7) There was no ADE in patients undergoing non-cardiac surgery with pacemaker dependency not programmed to an asynchronous pacing mode.
(8) We observed a significant postoperative impedance drop of all three leads potentially indicating anesthesia-related fluid overload, but further investigation is needed to evaluate the clinical relevance of this finding for the perioperative management of CRT patients.

### 4.1. Pre-Interventional Findings

Evaluating 172 pacemaker patients in 2004, Rozner et al. reported preoperative interventions in 15.7% of the cases [31]. A pre-interventional device malfunction requiring programming was documented in seven out of 60 pacemakers in another series including modification of pacing amplitude in three cases [28]. Since then, the continuous development of both hardware and software with the introduction of various often-automated tests and algorithms made interrogation and device function more reliable but also programming more challenging. In our population with complex systems, the anamnestic evaluation alone revealed device-associated adverse findings in 27%, including an increased LV-pacing threshold in 9.6%. The pre-interventional interrogation showed a high number of patients with an insufficient percentage of biventricular pacing and progredient cardiac decompensation in two cases. Out of 39 new device-related observations, 28 required an intervention. Most often, programming was necessary for increased LV pacing threshold (39%) to ensure proper device function. Although there was no need for intervention for vital indication, the high number of adverse findings underlines the relevance of a comprehensive preoperative clinical and technical examination in this particular population because the efficacy of CRT to improve chronic heart failure depends on optimal device programming to provide a maximum available amount of effective biventricular pacing.

### 4.2. Periprocedural Device-Related Adverse Events, Noise, and IME

Data on the incidence of EMI is limited, and most publications refer to case reports or small cohorts. In addition, there is a large difference between a high incidence of suspected events and confirmed clinically relevant EMI. In a recent evaluation of 2,940 ICD patients, the incidence of significant or potentially clinically significant EMI episodes was 0.27% per patient per year with nearly all in-hospital events occurring during cardiac surgery [27]. This contrasts with the data from the ICD-ON registry reporting episodes of nonphysiologic oversensing due to EMI in 34 out of 306 patients (11%) [20].

In our study, there was only one patient with four short VV intervals possibly due to monopolar electrosurgery near the device but no reported clinically relevant case of EMI. This is in line with the results of the recent PIM study, reporting no events of intraoperative EMI among 101 ICD patients [32].

Comparable with the observations from von Olshausen et al., there was no ADE in our population of CRT carriers undergoing non-cardiac surgery [27].

However, our data confirm an increased risk for relevant ADE after cardiac interventions performed in patients with complex biventricular CIEDs. For cardiac surgery and catheter ablations, maximum caution for manipulations near the leads is needed as well as a close and competent collaboration between the cardiologist, CIED specialist, anesthesiologist, and surgeon to prevent adverse events in these patients.

### 4.3. Sensor Programming

Rate-responsive sensors can be activated by EMI. To prevent sensor-mediated inappropriate tachycardia, it is recommended to suspend sensor function during surgery either by reprogramming or magnet application [17,33].

In our study, the sensor for rate adaptive pacing was deactivated routinely only for cardiac interventions. Among 100 CRT patients undergoing surgery with active rate responsive accelerometer sensor, there were no sensor-related perioperative ARE and there were no confirmed inappropriate sensor-mediated tachycardias. Therefore, given appropriate monitoring and information regarding the device programming, precautionary prophylactic sensor deactivation is not necessary for non-cardiac surgery.

### 4.4. Pacemaker Dependency

Pacemaker-dependent patients are supposed to be at higher risk in case of perioperative device dysfunction. To avoid EMI, the ESC guidelines on the management of noncardiac surgery 2014 recommended setting the device in an asynchronous or non-sensing mode in patients who are pacemaker-dependent [15]. According to the most recent 2021 ESC guidelines on cardiac pacing and CRT, magnet application should be preferred during electrocautery and the CIED reprogrammed to an asynchronous mode “if EMI is likely to occur or magnet stability cannot be guaranteed” in pacemaker-dependent subjects [33] (p. 3487). Data on the incidence of relevant events due to EMI in this population are rare. There was one case of brief pacing inhibition in a preoperatively non-dependent patient undergoing parathyroidectomy among 82 pacemaker-dependent subjects in the ICD-ON registry [20]. There are no data regarding the incidence of AE in pacemaker-dependent patients with active automatic function for sensing and automatic capture adjustment.

Among our population of CRT patients, eighty-one subjects were pacing-dependent. Only in one of them with increased RV pacing threshold, the device was programmed to VOO mode on request of the operator. All other patients underwent surgery without programming to an asynchronous or non-sensing mode, and at least one automatic algorithm for ventricular ACC was active in 26 cases (32.1%). In compliance with all precautions mentioned in Section 2, no significant intraprocedural asystole was reported in pacemaker-dependent CRT carriers, and therefore, routine prophylactic device reprogramming to asynchronous pacing appears inessential.

### 4.5. ICD Antitachycardia Function Suspension

The ESC guidelines on the management of non-cardiac surgery 2014 recommend deactivation of the defibrillator function either by reprogramming or magnet application [15]. The strategies were compared in the ICD-ON registry showing that no antitachycardia function suspension was needed in 69% of the CIEDs [20]. A safe and feasible strategy for ICD deactivation depending on the location of surgery and type of cautery used was described by Neubauer et al. [32]. In our study on CRT patients, the mode of deactivation was left to the decision of the responsible device specialist in close cooperation with the anesthesiologist and operator. Except for one patient who received inappropriate shocks of an ineffectively magnet-deactivated ICD for induced ventricular fibrillation during mitral valve surgery, there was no significant correlation between reprogramming and magnet application regarding the number and type of ARE or any change of electrode parameters. There was no inappropriate ICD therapy during magnet application for non-cardiac surgery. Further studies are needed to evaluate a more standardized approach, possibly even without deactivation under defined conditions in CRT-D patients.

### 4.6. Post-Interventional Interrogation

In 2004, Rozner et al. reported on a 4.7% incidence of postoperative pacemaker problems including one device reset and six pacing threshold increases (five right-ventricular, one atrial) in 149 surgical cases [31]. In our cohort, we observed no software reset but significant peri-interventional pacing threshold changes of at least one lead in 36% of CRT patients. Although there was no vital system damage, reprogramming was required in 16% of patients to ensure adequate device function, and lead revision was needed in two patients after cardiac surgery. Three out of the four ADEs in our series were detected only during postoperative device check. These data highlight the importance of carful post-interventional device interrogation in all patients with implanted CRT systems.

The intra-hospital remote monitoring could be extremely useful particularly in the post-operative phase, likely significantly reducing the need for the post-operative interrogation and thus lowering the work burden for the CIEDs team. This strategy should be investigated in further studies.

An interesting finding was a significant postoperative impedance drop of all three leads compared with preoperative values (Table 10). It has been shown that a decrease in the individual intrathoracic impedance correlates with an increased pulmonary capillary wedge pressure and volume overload because intrathoracic fluid accumulation due to pulmonary congestion improves electrical conductance [34]. In our population of patients with CRT devices implanted for chronic heart failure, it can be supposed that the observed significant impedance drop of all three electrodes reflects a postoperative anesthesia-related fluid overload. The clinical relevance of this finding for the perioperative management of CRT patients requires further evaluation.

### 4.7. Mortality

A seven-day mortality of up to 4% has been reported in patients undergoing non-cardiac surgery [35]. The ASA PS is a strong predictor of peri-interventional outcome [21,36]. Data from a large registry showed a death or severe complication rate of 26.2 per million anesthetic procedures in otherwise healthy ASA I and II subjects [21]. For patients with implanted CIED, the ASA PS is mostly III or higher, depending on the underlying disease. Twenty years ago, Samain et al. evaluated 73 pacemaker patients undergoing non-cardiac surgery reporting no significant device alterations but an 11% incidence of cardiac complications including an in-hospital mortality of 4.1% in mainly older subjects [37]. Another study reported on a 17% incidence of cardiac complications including two deaths among 65 patients with pacemaker undergoing non-cardiac surgery or invasive procedures, again without device dysfunction in these cases [28]. Data on the peri-interventional mortality of CRT carriers is rare. There were four post-procedural deaths (2%) in our study (Table 11) following one urgent and two emergency procedures. The mortality rate was substantial but related with the advanced/severe underlying disease in every case and in no patient associated with a CIED dysfunction.

## 5. Conclusions

In patients with implanted ICDs and pacemaker for CRT, comprehensive pre- and post-interventional device interrogation is mandatory to ensure proper perioperative system function. There was no device-associated mortality. For non-cardiac surgery, there was no intraoperative ADE, the type of antitachycardia function suspension in ICDs had no impact on a patient’s outcome, and most of the CRT-P devices did not require pre-interventional programming. Given the close cooperation between the CIED team, anesthesiologist, and operator, prophylactic activity sensor deactivation may not be necessary for non-cardiac interventions. Likewise, routine prophylactic device reprogramming to asynchronous pacing may be expendable.

## Figures and Tables

**Table 1:** Baseline characteristics.

All Patients	*n* = 198
Age (years)	72.2 ± 9.6
Age ≥ 80 years	47 (23.7%)
Gender, male	140 (70.7%)
CRT-ICD	134 (67.7%)
CRT-Pacemaker	64 (32.3%)
Pacemaker dependency	81 (41.1%)
Main indications for CRT	
Chronic heart failure	107/152 (70.4%)
Left bundle branch block	80/152 (52.6%)
Atrioventricular block	63/152 (41.4%)
Etiology of cardiomyopathy	
Ischemic	61/130 (47%)
Non-ischemic dilative	54/130(42%)
Other cardiomyopathies	15/130 (11%)
ICD implantation indication	
Primary prevention of SCD	97/120 (80.8%)
Secondary prevention of SCD	23/120 (19.2%)
LV-EF (%)	30.7 ± 9.5
LV-EF ≤ 35%	77 (79.4%)
% Biventricular pacing	90.4 ± 19.5
Heart rhythm prior to intervention	
Sinus rhythm	100/181 (55.2%)
Atrial fibrillation	74/181 (40.9%)
ASA PS	
ASA II	29 (14.7%)
ASA III	141 (71.6%)
ASA IV	27 (13.7%)
Neoplasia	35/192 (18.2%)
CRT Implant	
First CRT implanted device	124 (64.9%)
Previous PG replacement	51 (26.7%)
Previous upgrade from VVI/DDD to CRT	16 (8.4%)
Implantation Center	
Extern	114 (57.5%)
Klinikum Nuremberg	58 (29.3%)
Klinikum Fuerth (%)	26 (13.1%)

Values are mean ± SD if not stated otherwise. CRT = cardiac resynchronization therapy; ICD = implantable cardioverter-defibrillator; SCD = sudden cardiac death; LV-EF = left ventricular ejection fraction; ASA PS = American Society of Anesthesiologists physical status; PG = pulse generator

**Table 2:** Implanted devices and manufacturer.

Medtronic (*n*= 109)	Biotronik (*n* = 32)	SJM/Abbott (*n* = 27)	Guidant/BSCI (*n* = 30)
Amplia MRI Quad CRT-D DTMB2Q1 (3)	Enitra 8 HF-T QP (1)	Entrant HF CDHF A300Q (1)	Cognis 100-D P108 (3)
Amplia MRI CRT-D DTMBB2QQ (3)	Epyra 8 HF-T (1)	Epic HF V-339 (1)	Contac Renewal (2)
Brava CRT-D (6)	Evia HF-T (4)	Quadra Allure MP (2)	Contak Renewal 3 (1)
Brava Quad CRT-D (1)	Iforia 3 HF-T (3)	Quadra Allure MP RF 3262 CRT-P (1)	Contak Renewal 4 (6)
Cardia CRT-D (2)	Iforia 3 HF-T DF4 (1)	Quadra Assura (4)	Contak Renewal H 195 (1)
Compia MRI CRT-D (1)	Iforia 5 HF-T (8)	Quadra Assura 3367-40QC (7)	Contak Renewal TR 2 (2)
Compia MRI Quad CRT-D(4)	Iforia 7 HF-T (1)	Quadra Assura 3371-40QC (2)	Dynagen X4 CRT-D G158 (2)
Concerto C174 (3)	Intica 5 HF-T QP (1)	Unify 3235-40Q (6)	Incepta CRT-D P162 (3)
Consulta CRT-P C3TR01 (1)	Lumax 300 HF-T (1)	Unify Assura 3361-40QC (3)	Inogen CRT-D (1)
Egida CRT-D D394 TRG (2)	Lumax 340 HF-T (8)		Inogen X4 CRT-D G148 (3)
InSync 8040 (4)	Stratos LV-T (3)		Invive W173 (5)
InSync III 8042 (40)			Punctua CRT-D (1)
InSync III Marquis 7279 (6)			
InSync ICD 7272 (1)			
InSync Maximo 7304 (3)			
Protecta CRT-D (18)			
Syncra CRT-P (4)			
Viva Quad CRT-D (6)			
Viva XT CRT-D DTBA2D4 (1)			

Number of devices in brackets, in total 51 device types.

**Table 3:** **Preoperative CRT interrogation.** Observations and adverse findings already known from patient’s history or according to the device ID card in 39 patients.

	Observation/Adverse Finding	*n* = 43
**LV lead**	High pacing threshold	14
	Deactivated LV lead	5
	Low impedance	1
	Previous lead revision	3
	No lead	1
	Diaphragmatic stimulation	3
**RV lead**	High pacing threshold	4
	Low sensing	1
	Previous lead replacement	2
	Lead malfunction	2
**RA lead**	Undersensing of AF	1
	Low sensing	1
	Lead malfunction	1
	Failure automatic threshold algorithm with inappropriately high pacing output in RV/LV	1
	Two active devices implanted	2
	Safety warning for the system	1

LV = left ventricular; RV = left ventricular; RA = left atrial; AV = atrioventricular; AF = atrial fibrillation.

**Table 4:** **Preoperative CRT interrogation.** Observations and adverse findings in 180 patients requiring intervention in 22 subjects.

	Observation/Adverse Finding	*n* = 39	Action Taken	*n* = 28
**LV lead**	Increased pacing threshold	16	Increase output	9
	or loss of capture		Lead inactivated	2
	Oversensing	1	LV sensing deactivated	1
	VT induction during LV threshold test	2	VT ablation as scheduled	2
	Diaphragmatic stimulation	1	Change output	1
	Low Impedance	1		
**RV lead**	High pacing threshold	5	Increase output	5
	Oversensing	1	Decrease sense	1
	High % RV pacing (LV inactive)	1	AVD extended to avoid dyssynchrony	1
**RA lead**	High pacing threshold	1	Change output	1
	Oversensing	2	Decrease sense	1
	AF undersensing	3	Increase sense	1
			Change pacing mode	1
	SR undersensing	1		
	Lead malfunction	2	Change pacing mode	1
**Others**	ARI	1	Generator replacement post-OP	1
	nsVT during follow-up	1		

LV = left ventricular; RV = left ventricular; RA = left atrial; AVD = atrioventricular delay; VF = ventricular fibrillation; AF = atrial fibrillation; VT = ventricular tachycardia; nsVT = non-sustained VT; SR = sinus rhythm; ARI = anticipated replacement indicator.

**Table 5:** Anesthesiologic information of interventions above and below umbilicus.

	Location of Surgery		*p*-Value
	Above Umbilicus	Below Umbilicus	
All	*n* = 67	*n* = 130	
**Type of Surgery**			<0.000
Open surgery	51 (76.1%)	99 (76.1%)	
Endoscopic	2 (3.0%)	27 (20.8%)	
Catheter	12 (17.9%)	3 (2.3%)	
Laparoscopic	0	1 (0.8%)	
Microsurgery	2 (3.0%)	0	
**Urgency**			0.198
Elective	54 (80.6%)	103 (79.2%)	
Urgent	11 (16.4%)	15 (11.6%)	
Emergent	2 (3.0%)	12 (9.2%)	
**Type of electrocautery**			0.007
No electrocautery	7 (10.9%)	14 (11.7%)	
Monopolar	28 (43.8%)	73 (60.8%)	
Bipolar	21 (32.8%)	31 (25.8%)	
Others *	8 (12.5%)	2 (1.7%)	
**ASA PS**			0.190
II	14 (20.9%)	15 (11.5%)	
III	44 (65.5%)	97 (74.6%)	
IV	9 (13.6%)	18 (13.9%)	
**Ventilation**	44 (66.7%)	115 (88.5%)	<0.000
**Use of magnet**	19 (28.8%)	42 (32.6%)	0.628
**ICD therapies deactivated**	19 (35.2%)	41 (38.0%)	0.730

ASA PS = American Society of Anesthesiologists physical status. * Radiofrequency catheter ablation or electrocautery during colonoscopy.

**Table 6:** Indications for surgery or intervention.

Location of Surgery	Indication	N (%) Total 197
**Above umbilicus** ***n* = 67**	Heart disease	19 (28.3%)
	Neoplasia	13 (19.4%)
	Vascular disease	8 (11.9%)
	Infection/inflammation/abscess	8 (11.9%)
	CKD	5 (7.4%)
	Fracture	4 (6.0%)
	Eye disease	3 (4.5%)
	Orthopedic/surgical	2 (3.0%)
	Head disease/injury	2 (3.0%)
	Neurological disease	1 (1.5%)
	Thyroid disease	1 (1.5%)
	Tracheotomy	1 (1.5%)
**Below umbilicus** ***n* = 130**	Vascular disease	35 (26.9%)
	Fracture	22 (16.9%)
	Neoplasia	22 (16.9%)
	Gastrointestinal disease	11 (8.5%)
	Infection/inflammation	10 (7.7%)
	Orthopedic/surgical	9 (7.0%)
	Hernia	4 (3.1%)
	Urogenital disease	4 (3.1%)
	Hematoma	3 (2.3%)
	Neurological disease	3 (2.3%)
	Abscess	2 (1.5%)
	Spinal disease	2 (1.5%)
	Wound-healing disorder	2 (1.5%)
	CKD *	1 (0.8%)

* CKD = chronic kidney disease.

**Table 7:** Anesthesia-related events.

Stage of Surgery	Anesthesia-Related Events	Number of Events
**Preoperative** (26)	Hypotension	16
	Hypokalemia	4
	Coagulation disorder	2
	Difficult intubation	1
	Hypoglycemia	1
	Hypoxia	1
	Right heart failure	1
**Intraoperative** (60)	Hypotension	41
	Anemia	5
	Difficult intubation	3
	Hypokalemia	3
	Heart rate (not specified)	2
	Blood pressure (not specified)	1
	Cardiovascular system (not specified)	1
	Cardio-pulmonary resuscitation	1
	Diaphragmatic stimulation	1
	Ventricular arrhythmias terminated with ICD Shock	2
**Postoperative** (5)	Hypotension	2
	Unexpected extension of the surgery	1
	Heart rate (not specified)	1
	Paroxysmal atrial fibrillation	1

**Table 8:** Basic statistics for ARE and significant changes in the lead parameters.

General Population *n* = 198	ARE		*p*-Value	Any Significant Change Lead Parameter		*p*-Value
	Yes	No		Yes	No	
**Age**	74.9 ± 9.3	70.7 ± 9.6	0.004			
**Device**			0.849			0.421
** CRT-P**	23/69 (33.3%)	40/125 (32.0%)		24/62 (38.7%)	34/117 (29.1%)	
** CRT-D**	46/69 (66.7%)	85/125 (68.0%)		38/62 (61.3%)	83/117 (70.9%)	
**EF (%)**	27.5 ± 6.7	32.3 ± 10.4	0.022			
**%VP**	89.2 ± 21.9	90.7 ± 18.5	0.635			
**Preoperative follow-up**	66/69 (95.7%)	108/125 (86.4%)	0.042	60/62 (96.8%)	114/117 (97.4%)	<0.000
**History of syncope**	4/56 (7.1%)	5/99 (5.1%)	0.593	2/52 (3.8%)	7/99 (7.1%)	0.576
**Known preoperative abnormalities**	17/65 (26.2%)	22/116 (19.0%)	0.520	13/60 (21.7%)	24/111 (21.6%)	0.047
**New preoperative abnormalities**	27/54 (50%)	40/82 (48.9%)	0.255	24/44 (54.5%)	40/84 (47.6%)	<0.000
**PM-dependent**	34/69 (49.3%)	45/124 (36.3%)	0.077	28/62 (45.2%)	50/117 (42.7%)	0.026
**Escape rhythm > 30 bpm**	9/31 (29.0%)	9/47 (19.1%)	0.311	6/27 (22.2%)	11/50 (22.0%)	0.900
**Sensor initial**			0.166			0.477
** Off**	26/69 (37.7%)	60/125 (48.0%)		24/62 (38.7%)	54/117 (46.2%)	
** On**	43/69 (62.3%)	65/125 (52.0%)		38/62 (61.3%)	63/117 (53.8%)	
**Any reprogramming preoperative**	31/69 (44.9%)	52/125 (41.6%)	0.009	24/62 (38.7%)	59/117 (50.4%)	<0.000
**Reprogramming sensor preoperative**			0.031			0.701
** Sensor active**	36/42 (85.7%)	63/65 (96.9%)		36/38 (94.7%)	56/62 (90.3%)	
** Sensor deactivated**	6/42 (14.3%)	2/65 (3.1%)		2/38 (5.3%)	6/62 (9.7%)	
**Sensor during OP**			0.813			0.320
** Sensor on**	36/69 (52.2%)	63/125 (50.4%)		36/62 (58.1%)	56/117 (47.9%)	
** Sensor off**	33/69 (47.8%)	62/125 (49.6%)		26/62 (41.9%)	61/117 (52.1%)	
**Rhythm before intervention**			0.217			<0.000
** SR**	38/66 (57.6%)	60/111 (54.1%)		34/62 (54.8%)	65/116 (56.0%)	
** AF**	27/66 (40.9%)	45/111 (40.5%)		25/62 (40.3%)	45/116 (38.8%)	
**Neoplasia**	9/68 (13.2%)	26/121 (21.5%)	0.161	8/60 (13.3%)	26/113 (23.0%)	0.089
**Surgery type**			0.501			0.640
** Above umbilicus**	21/69 (30.4%)	44/125 (35.2%)		24/62 (38.7%)	37/116 (31.9%)	
** Below umbilicus**	48/69 (69.6%)	81/125 (64.8%)		38/62 (61.3%)	79/116 (68.1%)	
**Urgency**			0.157			0.100
** Elective**	54/69 (78.3%)	100/125 (80.0%)		45/62 (72.6%)	97/117 (82.9%)	
** Urgent**	7/69 (10.1%)	19/125 (15.2%)		12/62 (19.3%)	14/117 (12.0%)	
** Emergent**	8/69 (11.6%)	6/125 (4.8%)		5/62 (8.1%)	6/117 (5.1%)	
**ASA PS**			0.787			0.115
** II**	9/69 (13.0%)	20/124 (16.1%)		7/61 (11.5%)	17/117 (14.5%)	
** III**	51/69 (74.0%)	86/124 (69.4%)		42/61 (68.8%)	89/117 (76.1%)	
** IV**	9/69 (13.0%)	18/124 (14.5%)		12/61 (19.7%)	11/117 (9.4%)	
**Ventilated**	67/69 (97.1%)	90/125 (72.0%)	<0.000	52/62 (83.9%)	92/115 (80.0%)	0.884

ARE = anesthesia-related event; CRT-P = cardiac resynchronization therapy-pacemaker; CRT-D = cardiac resynchronization therapy-defibrillator; EF = ejection fraction; VP = ventricular pacing; PM = pacemaker; SR = sinus rhythm; AF = atrial fibrillation; ASA PS = physical status defined by the American Society of Anesthesiologists; ICD = implantable cardioverter-defibrillator.

**Table 9:** **Post-operative CRT interrogation.** Observations requiring intervention in 29 patients.

	Observation/Adverse Finding	Action Taken *	*n* = 33
**LV lead**	Increased pacing threshold	Increase output	12
		Change pacing polarity	1
		Change pacing mode	2
		Lead inactivated	2
	Loss of capture	System/lead revision	2
	Lower pacing threshold	Decrease output	3
**RV lead**	Increased pacing threshold	Increase output	2
**RA lead**	Undersensing of AF	Increase sensing	2
**Lower rate limit**	Regarded as inappropriate low	Increase LRL	3
	Regarded as inappropriate high	Decrease LRL at night	3
**AV delay**	AV dyssynchrony	AV delay optimized	1

* Some of the reprogrammings were performed as a consequence of observations already made during preoperative interrogation. LV = left ventricular; RV = left ventricular; RA = left atrial; AV = atrioventricular; AF = atrial fibrillation.

**Table 10:** Specific CRT lead parameters in pre- and postoperative follow-up.

Lead	Pre OP	Post OP	*p* Value	Frequency of Detected Significant Change
All *				**64/179 (35.8%)**
**RA**				
Sensing (mV)	2.75 ± 1.78	2.37 ± 1.43	0.000	16/129 (12.4%)
Pacing threshold (V)	0.73 ± 0.31	0.79 ± 0.75	0.410	11/100 (11%)
Pacing threshold (msec)	0.41 ± 0.13	0.41 ± 0.13	0.540	
Impedance (Ohm)	566 ± 536	559 ± 564	0.000	11/136 (8.1%)
**RV**				
Sensing (mV)	12.2 ± 5.4	11.5 ± 5.18	0.010	15/150 (10%)
Pacing threshold (V)	0.78 ± 0.34	0.77 ± 0.35	0.784	11/175(6.3%)
Pacing threshold (msec)	0.42 ± 0.11	0.43 ± 0.14	0.675	
Impedance (Ohm)	576 ± 188	549 ± 184	0.000	11/180 (6.1%)
**LV**				
Sensing (mV)	13.6 ± 6.6	13.3 ± 6.5	0.023	17/62 (27%)
Pacing threshold (V)	1.37 ± 0.98	1.41 ± 1.19	0.334	11/166 (6.6%)
Pacing threshold (msec)	0.55 ± 0.29	0.55 ± 0.29	0.098	
Impedance (Ohm)	645 ± 249	615 ± 238	0.000	19/170 (11.2%)

Values are mean ± SD. *p*-values by Student’s *t*-tests. * At least one lead with significant change in a device.

**Table 11:** Peri-interventional mortality in CRT carriers.

	Age	LV-EF	CIED	PM Dependency	Observation Pre Surgery	Programming Pre Surgery	Type of Surgery	Urgency of Surgery	ASA PS	Electro-Cautery	ARE	**ADE**	**Cause of Death**	**Time Surgery to Death**
1	84		CRT-P	no		0	Femoral neck fracture	Urgent	3	Mono	0	0	Acute renal failure	12 days
2	88		CRT-P	yes		Sensor off	TAVI, transapical	Elective	4	Mono	CPR	0	Cardiogenic shock	9 days
3	74	15%	CRT-D	no		0	Intestinal neoplasm, acute ischemia	Emergency	4	Mono	0	0	Acute renal failure, cardiogenic shock	<24 h
4	77	20%	CRT-D	yes	Cardiac decompensation, High ventricular pacing thresholds	0	Acute occlusion subclavian and carotid artery	Emergency	3	Mono	Hypotension	0	Bihemispheric infarction, malignant edema	One day

LV-EF = left ventricular ejection fraction; CIED = cardiac implanted electronic device; ASA PS = American Society of Anesthesiologists physical status; ARE = anesthesia-related observation/event; ADE = adverse device-related event; CRT-P = cardiac resynchronization therapy pacemaker; CRT-D = cardiac resynchronization therapy ICD; TAVI = transcatheter aortic valve implantation; CPR = cardiopulmonary resuscitation.

## Data Availability

Data can be provided on reasonable request by the corresponding authors.
